# BONE FRAGILITY, FRACTURE RISK AND TRAUMA:A COMPLICATED TRIANGLE IN CHILDREN

**DOI:** 10.1590/1413-785220172502163455

**Published:** 2017

**Authors:** De-fa Huang, Deng-kun Lv, Qi-lin Zhao, Li-feng Zhang

**Affiliations:** 1 Department of Pediatrics Surgery, Shandong Jining No.1 People's Hospital, Jining, Shandong 272111, China

**Keywords:** Fractures, bone/epidemiology, Bone density/physiology, Child, Risk factors

## Abstract

**Objective:**

To analyze whether association between bone fragility and risk of fracture depends on the trauma level.

**Method:**

All participants along with their mothers underwent DXA scan and body measurements. The subjects answered a self-report questionnaire about their physical activities and the precipitating causes. The questionnaire results were associated with DXA performed at the baseline visit.

**Results:**

A total 374 children with available DXA scan and complete follow-up of 5 years were included in the final analysis. Of the 374 children, 53 (14.2%) had one fracture, and 11 (20.7%) had more than one fracture. Based on the modified Landin classification, the trauma level was determined. Of the 53 (14.2%) children who had one fracture, 39 (73.6%) were classified, namely 19 (48.7%) with mild trauma, 16 (41%) with moderate trauma and four (10.2%) with severe trauma. Trauma level could not be assigned to 14 (26.4%) children due to limited information. Children without fractures had significantly higher values in all bone parameters compared to those with fractures caused by mild trauma.

**Conclusion:**

Subjects with mild trauma fractures had an inversely proportional ratio between bone fragility parameters and fracture risk compared with subjects without fractures. ***Level of Evidence IV, Case Series.***

## RESUMO

**Objetivo:**

Analisar se a associação entre fragilidade óssea e risco de fratura depende do nível de trauma.

**Método:**

Todos os participantes, juntamente com suas mães, foram submetidos à DEXA e medições corporais. Os participantes responderam um questionário de autorrelato sobre atividades físicas e descrição de como o trauma ocorreu. Os resultados do questionário foram associados à DEXA realizada na primeira visita do estudo.

**Resultados:**

Um total de 374 crianças com DEXA disponível e acompanhamento completo de 5 anos foi incluído na análise final. Das 374 crianças, 53 (14,2%) tiveram uma fratura e 11 (20,7%) tiveram mais de uma fratura. Com base na classificação de Landin modificada, foi determinado o nível de trauma. Das 53 (14,2%) crianças que tiveram uma fratura, 39 (73,6%) foram classificadas, sendo 19 (48,7%) com trauma leve, 16 (41%) com trauma moderado e quatro (10,2%) com trauma grave. O nível de trauma não pôde ser atribuído a 14 (26,4%) crianças, devido a informações limitadas. As crianças sem fraturas apresentaram valores significantemente mais altos em todos os parâmetros ósseos, em comparação com os que tinham fraturas causadas por trauma leve.

**Conclusão:**

Os indivíduos com fraturas por trauma leve apresentaram relação inversamente proporcional entre os parâmetros fragilidade óssea e o risco de fratura em comparação com indivíduos sem fratura. ***Nível de Evidência IV, Série de Casos*** .

## INTRODUCTION

A bone fracture is defined as damage in the continuity of bone. Analysis of data from many studies has shown that fractures comprise 10-25% of childhood trauma.^1^ Despite this high prevalence, healthcare professionals and public health programs tend to focus on the adult population. Epidemiological studies have shown that the incidence of fractures during childhood is somewhat similar to the incidence of fractures in the elderly population.^2^ ^,^ ^3^

It is a well-established fact that low bone mineral density and a previous history of fracture are the strongest risk factors for future fractures.^4^ ^,^ ^5^ However, it is assumed the relationship between the history of fracture and risk of future fracture is strongest for fractures that occur as a consequence of low trauma, such as falls from an upright position.^6^ During evaluation of risk factors for osteoporosis and other bone diseases, history of childhood fractures is generally ignored on the assumption that childhood fractures are primarily caused by high trauma.^7^ ^-^ ^9^ Bone fragility that might cause both high and low trauma fractures is itself not considered a risk for future fractures.^10^ However, there is emerging evidence that childhood fractures are associated with underlying skeletal fragility.^11^ ^,^ ^12^ A meta-analysis of case control studies by Clark et al.^13^ ^)^ showed an inverse relationship between bone mineral density and risk of fracture during childhood. These findings are also supported by other observational studies.^14^ ^,^ ^15^ ^)^ The Avon Longitudinal Study of Parents and Children (ALSPAC) observed an inverse association between volumetric bone mineral density (vBMD) and fracture risk.^14^ The overall results of these studies indicated that childhood fractures are associated with underlying skeletal fragility. 

Landin^16^ ^)^ derived a classification system defining different levels of trauma in children based on the events that preceded injuries or fractures in his study population. The three main components of his classification system were height of fall, type of activity, and any device that may have resulted in the fall; these components were used to categorize trauma into three different levels (slight, moderate, and severe trauma, respectively). Using a modified version of Landin's classification system,^10^ we conducted a prospective cross-sectional study to examine whether association between bone fragility and fracture risk depends on the level of trauma preceding injury. Landin's modified trauma levels used in the current study are as follows: low trauma includes falling and landing on the ground (<0.5 m) or a resilient surface (0.5-3 m), falling from a bed/sofa/cot, injuries sustained during play on the playground or low-impact sports such as gymnastics, judo, etc. Moderate trauma injuries include falling and landing on a non-resilient surface (0.5-3m), falling from a bicycle, skateboard, swing, slide, rollerblades, or bunkbed. High/severe trauma includes falling from >3 m, traffic accidents, and being hit by a heavy moving object.

## METHODS

A total of 457 healthy children (mean 10.1 years of age) visiting Shandong Jining No.1 People's Hospital were included in the current study. This study was approved by the ethics committee of Shandong Jining No.1 People's Hospital (approval number : ZK242403). The majority (64.7%) were female. Children with malnutrition, chronic diseases, or any history of bone disease or mal-absorption were excluded. We also excluded children who took medications regularly or who had been prescribed calcium and vitamin D supplements. The study was approved by the institutional review board. All participating children gave verbal consent, while the guardians signed a term of free and informed consent. All participating children were invited to come to the clinic accompanied by their mothers to undergo DXA scanning and further measurement of height, weight, body mass index

(BMI, kg/m^2)^, and collection of other basic demographic data. Based on the recommendations from the International Society for Clinical Densitometry (ISCD), instead of full-body DXA scans we used the total body less head (TBLH) bone area (BA) and TBLH bone mineral content (BMC) in the current study.^(17)^ In children, TBLH is recommended for its accuracy and precise results. We also opted not to use the full-body DXA scan because the head is not receptive to stimuli

(such as exercise).^(18)^ Height was measured with subject standing straight with feet flat on the ground and heels touching the back plate of the measuring instrument. Height was measured to the last millimeter (mm) while weight was measured to the nearest 50 g. Measurement of TBLH BA and TBLH BMC was taken with a Lunar Prodigy DXA device. The precision of the DXA scan was expressed in terms of coefficient of variation (CV), i.e. 0.8%. The CV value is based on 150 repeated scans. 

After measurement of the physical parameters, the subjects took a self-reported questionnaire inquiring about their participation in physical activities such as dancing, running, swimming, aerobics, etc. and the amount of time they engaged in such activities per week. Puberty was assessed using the Tanners and Whitehouse classification of breast development and pubic hair^19^ ^)^ through drawings. Parental race, educational qualification, and social status were noted by a researcher on a self-reported questionnaire.

To collect information on fracture incidence and description of events surrounding the injury, subjects were given a self-reported questionnaire at their each yearly follow-up visit for a 5-year period. These results were then linked with the subjects' DXA scan, which was performed during their first visit when the study began. Subjects who reported a fracture were then asked to fill out another questionnaire collecting information about the nature of the injury and preceding circumstances. Finally, in order to confirm fracture, the subjects' parents were asked to provide the X-ray report, if this was available. When the X-ray report was not available, "not confirmed fracture" was used as an outcome. Additional data was collected using a modified version of Landin's classification. 

The demographic data were presented as mean and frequencies when appropriate. The two-tailed Student's t-test was used to find significant differences between children with and without fractures. In order to determine bone variables in children who had fractures and those without fractures, we ran a linear regression model, using both unadjusted and adjusted models (age, sex, race, economic status). All statistical analysis was done using SPSS (Statistical Package for Social Sciences) software version 20.

## RESULTS

**Figure 1:**
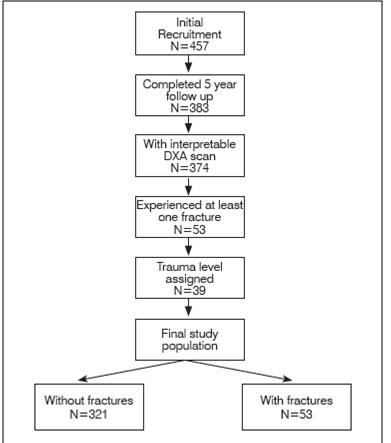
Methodological flowchart of study.

Of the 457 healthy children initially recruited for the study, 383 children completed the 5 year follow-up. Of the 383 DXA scans, 9 scans could not be interpreted, yielding a total of 374 available scans of the children with complete follow-up who were included in the study (Figure 1). The demographic and clinical profiles of the children who had fractures and did not have fractures are shown in Table 1. Of the total population of 374 followed for 5 years, 53 (14.2%) sustained at least one fracture, and of this group 11 (20.7%) experienced more than one fracture. Using the modified Landin trauma classification, we assigned a trauma level in 39 (73.6%) of the 53 children who reported a fracture: 19 cases of slight trauma (48.7 %), 16 cases of moderate trauma (41%), and 4 cases of severe trauma (10.2%). Trauma level could not be assigned in 14 cases (26.4%) due to limited information from both parents and children regarding the incidents preceding the fractures. 

**Table 1:** Demographic profile of study subjects.

Variable	Subjects without fracture (N=321)	Subjects with fracture (N=53)	p-value
Age (years)	10.2 ± 0.4	10.2 ± 0.3	0.347
Male sex	158 (49.2%)	28 (52.8%)	0.251
Height (cm)	138.2 ± 6.3	138.6 ± 5.9	0.614
Weight (kg)	35.4 ± 6.4	36.0 ± 8.3	0.315
BMI (kg/m^2)^	17.1 ± 2.4	17.9 ± 3.9	0.041
Socioeconomic status			
Low	109 (33.9%)	17 (32.0%)	0.06
Moderate to high	212 (66.1%)	36 (68%)	
Parental education			
School	87 (27.2%)	15 (28.3%)	0.328
College	135 (42.0%)	22 (41.5%)	
University	99 (30.8%)	16 (30.1%)	
Tanner staging			
1	161 (50.1%)	27 (50.9%)	0.158
2	146 (45.4%)	23 (43.3%)	
>3	14 (4.3%)	3 (5.6%)	
Weekly physical activity			
<3 times	167 (52%)	19 (35.9%)	0.024
>3 times	154 (48%)	34 (64.1%)

**Figure 2:**
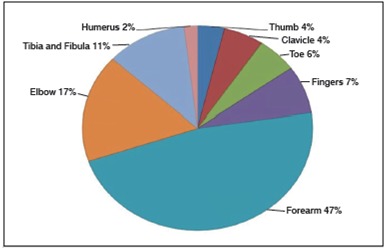
Types of fractures reported.

Of the 53 (14.2%) children who experienced a fracture, the majority of the fractures were reported to be in the forearm, with 25 cases (47%), while the least-reported fracture site was the humerus, with 1 case (2%). Other reported fracture sites were the elbow, with 9 cases (17%), the tibia and fibula with 6 cases (11.3%), the fingers with 4 cases (7.5%), the toe with 3 cases (6%), the clavicle with 3 cases (5.6%), and the thumb with 2 cases (4%) (Figure 2).

Without adjusting for variables, we compared demographic data as well as different bone parameters in children with fractures and those without fractures. (Table 1) Based on this analysis, except for BMI and weekly level of physical activity there was no significant difference in the demographic variables of the study subjects. As for bone parameter variables, subjects who did not experience fractures had a statistically significant increase in humeral volumetric density (cm^3)^ while no difference was seen in the other parameters

(TBLH BMC, TBLH BA, TBLH BMD). To pursue more detailed analysis, we further subdivided the children with fractures according to trauma level. Because the number of cases with fracture caused by severe trauma were so low, for this analysis we combined fractures with moderate and severe levels of trauma and compared them with fractures caused by low trauma level and no fractures. (Table 2) Children without fractures had statistically significant higher values for all bone parameters compared to children who experienced fractures caused by slight trauma. However, only humeral vBMD and TBLH bone size relative to body size was lower in children with fractures caused by high levels of trauma, while statistically significant decreases were not seen in other parameters. We also performed an adjusted analysis (age, sex) and found a similar reduction in humeral vBMD in all the children who experienced fractures compared with those without fractures. Finally, subjects with low trauma fractures weighed less (approximately 7%) and subsequently had lower BMIs than their counterparts who experienced high or moderate trauma fractures. Moreover, subjects with low trauma fractures also had lower BMD, BA AND TBLH BMC values than those subjects who had high or moderate trauma fractures.

**Table 2:** Difference in bone parameters of subjects without fractures and subjects with fractures categorized by trauma level.

Bone parameter	Subjects without fracture N=321	Subjects with fracture N=53	p-value *	Subjects with low trauma fractures N=19	p-value #	Subjects with high trauma fractures N=20	p-value ^
TBLH BMC (g)	889 ± 187	881 ± 175	0.416	845 ± 156	0.002	901 ± 179	0.425
TBLH BA (cm^2)^	1132 ± 163	1129 ± 149	0.384	1101 ± 135	0.014	1147 ± 158	0.342
TBLH BMD (g/cm^2)^	0.775 ± 0.053	0.771 ± 0.051	0.058	0.759 ± 0.051	0.004	0.774 ± 0.054	0.572
Humeral vol. density	0.489 ± 0.05	0.473 ± 0.05	<0.001	0.471 ± 0.05	<0.001	0.469 ± 0.05	0.003

*p-value: for difference between children without fractures and children with fractures. #p-value: for difference between children without fractures and children with low trauma fractures. ^p-value: for difference between children without fractures and children with high trauma fractures. All statistically significant values (p-value >0.05) are shown in bold. BMC: bone mineral content, BA: bone area, BMD: bone mineral density, TBLH: total body less head.

## DISCUSSION

To the best of our knowledge, this is the first prospective study following Chinese children over a long follow-up period (5 years) that evaluates the relationship between future fracture risk and underlying skeletal fragility and whether this relationship is influenced by trauma level. The incidence of fracture in our study cohort was 14.2%, which is comparable to previously conducted studies. Clark et al. followed 7725 children in a community over a span of 2 years and found an overall fracture incidence rate of 8.9%.^10^

Our findings showed that overall fracture risk is greater in boys and low trauma fractures are more common in children. Increased fracture risk in boys has been reported in many previously conducted studies and is attributed to their behavior and restless nature.^20^ ^,^ ^21^ Regardless of the trauma level preceding fracture, children with fracture had reduced bone parameters such as humeral vBMD, TBLH bone size relative to body size, and TBLH vBMD. These findings are consistent with other prospective and case controlled studies which showed similar results in children belonging to different age groups.^10^ ^,^ ^15^ ^,^ ^22^

Our main focus in this paper was to observe the association between skeletal fragility and fracture risk and trauma level. We found that subjects with low trauma fractures had an inverse relationship between bone fragility parameters (TBLH vBMD, humeral vBMD, TBLH bone size relative to body size) and fracture risk compared with subjects who did not have fractures. Interestingly, with the exception of TBLH vBMD, other bone fragility parameters (humeral vBMD, TBLH bone size relative to body size) were also inversely related to fracture risk in children with moderate to severe trauma before fracture. Although more skeletal fragility is observed in subjects with low trauma, we conclude that skeletal fragility in early life is related to future fracture risk even at high trauma levels.In contrast, we have observed that among subjects with fractures the bone parameters such as TBLH BMC and TBLH vBMD are reduced compared to those subjects without fractures. This discrepancy in results can be explained by the fact that majority of fractures in our study affected the upper limb. Compared to the whole body, upper limb fractures are weakly related to skeletal fragility parameters. 

We unexpectedly found weight differences among subjects with low and high trauma levels. Subjects with high trauma fractures had more fat and lean mass and consequently higher BMIs than children with low trauma fractures. Contrary to the observation that obese adults are at less risk for osteoporosis,^23^ ^,^ ^24^ increased weight is a risk factor for fractures in children.^11^ ^,^ ^25^ It has been proposed that overweight children have low bone area for their weight, placing them at high risk for fracture.^26^ ^,^ ^27^ Our results showed that subjects with high trauma fractures had higher TBLH and BMC values than subjects with low trauma fractures. On the other hand, bone size relative to body size was reduced in both high trauma and low trauma fractures, suggesting that higher values for bone parameters

(TBLH BA and BMC) in overweight children cannot compensate for their increased body weight. Lastly, despite trauma level, fracture risk in childhood was associated with level of physical activity in our study cohort. Subjects with active or rigorous participation in physical activities reported more fractures. These findings are consistent with previously reported studies.^28^ ^,^ ^29^ MA and Jones conducted population based case control studies to evaluate the risk of upper limb fractures and physical activity and found similar results.^28^

The main limitation associated with the current study is self-categorization of trauma level and self-reported responses by the participants in different questionnaires. Since questionnaires were not given to participants immediately after the fracture occurred but rather some time later during the scheduled follow-up meeting, recall bias cannot be ignored. Moreover, trauma level was not assigned in all participants and not all reported fractures were confirmed by X-ray reports. Despite these limitations, the current study is strengthened by its prospective nature and long follow-up period. Moreover, the DXA scans were done at the beginning of the study before the fractures occurred, assuring that the scan results were not be influenced by the fracture. Lastly, the drop-out rate in our study was quite low, permitting generalized results. 

## CONCLUSION

The current study conclusively confirms the proposed hypothesis that regardless of trauma level preceding the injury, skeletal fragility contributes to fracture risk in children. Further longitudinal observational studies are needed to explore whether this risk is transient or remains persistent. Furthermore, future studies should observe the influence of skeletal fragility on fracture risk in both elderly men and women. Because fractures are among the most important clinical and public health concerns in both adults and children, future studies should target both populations.
